# Genomic profiling of murine mammary tumors identifies potential personalized drug targets for p53-deficient mammary cancers

**DOI:** 10.1242/dmm.025239

**Published:** 2016-07-01

**Authors:** Adam D. Pfefferle, Yash N. Agrawal, Daniel C. Koboldt, Krishna L. Kanchi, Jason I. Herschkowitz, Elaine R. Mardis, Jeffrey M. Rosen, Charles M. Perou

**Affiliations:** 1 Department of Pathology and Laboratory Medicine, University of North Carolina, Chapel Hill, NC 27599, USA; 2 Lineberger Comprehensive Cancer Center, University of North Carolina, Chapel Hill, NC 27599, USA; 3 The McDonnell Genome Institute, Washington University School of Medicine, St Louis, MO 63108, USA; 4 Department of Biomedical Sciences, University at Albany, Rensselaer, NY 12144, USA; 5 Department of Molecular and Cellular Biology, Baylor College of Medicine, Houston, TX 77030, USA; 6 Department of Genetics, University of North Carolina, Chapel Hill, NC 27599, USA

**Keywords:** Basal-like breast cancer, Exome sequencing, Genetically engineered mouse models, p53, Personalized genomics, Whole-genome sequencing

## Abstract

Targeted therapies against basal-like breast tumors, which are typically ‘triple-negative breast cancers (TNBCs)’, remain an important unmet clinical need. Somatic *TP53* mutations are the most common genetic event in basal-like breast tumors and TNBC. To identify additional drivers and possible drug targets of this subtype, a comparative study between human and murine tumors was performed by utilizing a murine *Trp53-*null mammary transplant tumor model. We show that two subsets of murine *Trp53-*null mammary transplant tumors resemble aspects of the human basal-like subtype. DNA-microarray, whole-genome and exome-based sequencing approaches were used to interrogate the secondary genetic aberrations of these tumors, which were then compared to human basal-like tumors to identify conserved somatic genetic features. DNA copy-number variation produced the largest number of conserved candidate personalized drug targets. These candidates were filtered using a DNA-RNA Pearson correlation cut-off and a requirement that the gene was deemed essential in at least 5% of human breast cancer cell lines from an RNA-mediated interference screen database. Five potential personalized drug target genes, which were spontaneously amplified loci in both murine and human basal-like tumors, were identified: *Cul4a*, *Lamp1*, *Met*, *Pnpla6* and *Tubgcp3*. As a proof of concept, inhibition of *Met* using crizotinib caused *Met-*amplified murine tumors to initially undergo complete regression. This study identifies *Met* as a promising drug target in a subset of murine *Trp53*-null tumors, thus identifying a potential shared driver with a subset of human basal-like breast cancers. Our results also highlight the importance of comparative genomic studies for discovering personalized drug targets and for providing a preclinical model for further investigations of key tumor signaling pathways.

## Abstract (precis)

**Editors' choice:** Personalized therapeutic targets for triple-negative breast cancers remain an important unmet clinical need. Genomic profiling of murine *Trp53*-null tumors identified a number of promising drug targets conserved in humans.

## INTRODUCTION

Human breast cancer is a heterogeneous disease that can be segregated into at least six distinct subtypes based on gene expression profiles: basal-like, claudin-low, human epidermal growth factor receptor 2 (HER2)-enriched, luminal A, luminal B and normal-like (Perou et al., 2000; Prat et al., 2010; Cancer Genome Atlas Network, 2012). Although targeted therapeutics exist for estrogen receptor (ER)-positive (Jordan, 2003) [luminal A/B (Prat and Perou, 2011)] and HER2-positive (Hynes and Lane, 2005) [HER2-enriched (Prat and Perou, 2011)] tumors, targeted treatments for triple-negative breast cancer (TNBC) [basal-like and claudin-low (Prat and Perou, 2011)] remain an important unmet clinical need (Carey et al., 2010). To address this, a research emphasis has been placed on identifying the molecular drivers of basal-like and claudin-low tumors that could be exploited as drug targets for these subtypes.

Somatic *TP53* mutations are one of the most frequent genetic events in breast cancer, occurring in >80% of TNBCs (Cancer Genome Atlas Network, 2012). Although there is a growing appreciation for the consequences that *TP53* gain-of-function mutations impose on cell signaling (Brosh and Rotter, 2009; Murphy et al., 2000), the majority of these TNBC *TP53* mutations are predicted to lead to loss of function (Bullock and Fersht, 2001). This genetic foundation primes tumors to accumulate secondary genetic aberrations by decreasing the cell's ability to maintain normal cell physiology (Murphy and Rosen, 2000; Hanel and Moll, 2012). Identifying the subset of genetic events that promote breast cancer is important for informing tumor biology and for guiding personalized treatment regimens. However, segregating genetic drivers of tumorigenesis from passenger aberrations is inherently difficult owing to the diversity of breast tumors and the large number of candidate aberrations identified in genome-wide profiling studies (Cancer Genome Atlas Network, 2012; Curtis et al., 2012).

Comparative studies between human and murine tumors provide an attractive approach for narrowing the genetic-driver candidate list by highlighting conserved features between species (Pfefferle et al., 2013; Silva et al., 2015). The murine *Trp53*-null mammary transplant model (Jerry et al., 2000) is a particularly powerful resource for identifying the genetic drivers of TNBC. From a genetics perspective, the *Trp53*-null transplant model mimics the loss of function seen in human tumors through the expression of a truncated version of *Trp53* (Jacks et al., 1994). In addition, tumors from this model resemble multiple human intrinsic subtypes of breast cancer, including both basal-like and claudin-low (Herschkowitz et al., 2012; Pfefferle et al., 2013). Identifying the genetic mechanisms that explain this intra-model tumor heterogeneity might help in determining the etiology of specific human subtypes. From an experimental perspective, the transplantability of these tumors allows for a single tumor to be expanded and exhaustively studied to verify that the conserved candidates are drivers of tumorigenesis and/or to rigorously test therapeutics (Roberts et al., 2012a,b; Usary et al., 2013; Bennett et al., 2012). For these reasons, this study used the *Trp53*-null mammary transplant model to identify genetic drivers, and thus potential drug targets, of basal-like breast tumors.

## RESULTS

### *Trp53*-null transplant tumors are counterparts for multiple expression subtypes of human breast cancer

**Fig. 1.**
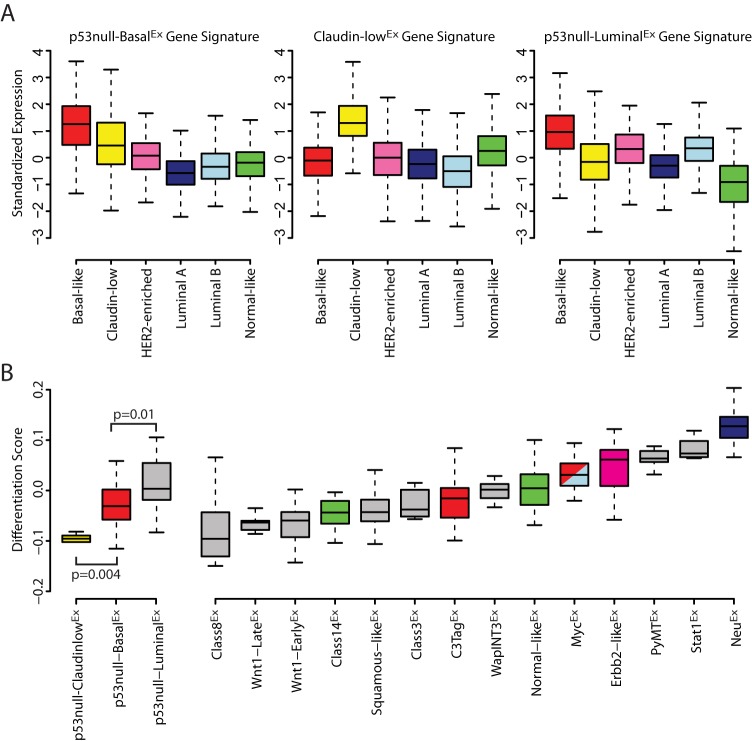
**Human counterparts of *Trp53*-null transplant tumors.** (A) Genes highly expressed within each *Trp53*-null transplant class were identified using a two-class (class *x* versus all others) SAM analysis (FDR 0%) across our 385-sample murine microarray dataset. The standardized average of these gene signatures was calculated across more than 3000 human tumors and displayed by intrinsic subtype. (B) Tumor differentiation scores (D-Scores) (Prat et al., 2010) were calculated for all 385 murine samples and displayed by intrinsic class. The D-Scores of the three *Trp53*-null transplant classes were compared using a Student's *t*-test.

The *Trp53*-null transplant model produces phenotypically and genomically diverse tumors that can be classified into three major subtypes/classes based on gene expression profiles: p53null-Basal^Ex^, Claudin-low^Ex^ and p53null-Luminal^Ex^ (Pfefferle et al., 2013). A crucial component of breast cancer comparative studies is to properly identify corresponding human-to-murine subtype counterparts. Once counterparts are determined, conserved features can be identified to highlight the candidate genetic drivers of those subtypes. For this purpose, a transcriptomic comparison between the two species was performed. To do this, we created gene signatures for each of our three previously identified *Trp53*-null transplant classes using a two-class (class x versus all others) Significance Analysis of Microarrays (SAM) analysis across a 385-sample microarray dataset consisting of 27 murine models of mammary carcinoma, and normal mammary tissue (Pfefferle et al., 2013). Each signature was defined as all genes highly expressed in the class of interest with a false discovery rate (FDR) of 0%. The average of these signatures was calculated within each sample of the UNC308 (Prat et al., 2010), Combined855 (Harrell et al., 2012) and Metabric (Curtis et al., 2012) human breast cancer datasets to identify which human tumors also highly expressed these same sets of genes. As anticipated from previous human subtype associations (Pfefferle et al., 2013), the murine p53null-Basal^Ex^ subtype signature was highly expressed in basal-like human tumors and the Claudin-low^Ex^ signature was specific to the human claudin-low subtype (Fig. 1A). Although the p53null-Luminal^Ex^ signature was most highly expressed in basal-like human tumors, it was also highly expressed in HER2-enriched and luminal-B tumors, indicating that these murine tumors have expression features in common with several human subtypes. Similar results were observed when we compared 963 molecular-pathway-based signatures across the three murine subtypes (Fig. S1) (Pfefferle et al., 2013).

One explanation for the transcriptomic associations observed in Fig. 1A is that both the human and murine subtypes arise from similar cell types within the mammary-gland hierarchy (Visvader, 2009). To address this possibility, a ‘differentiation score’ (D-Score) was calculated for all tumors in the murine microarray dataset (Fig. 1B) (Prat et al., 2010). Low scores indicate a tumor similarity to adult mammary stem cells (aMaSCs), intermediate scores a similarity to luminal progenitor (LumProg) cells, and high scores a similarity to mature luminal (MatureLum) cells (Prat et al., 2010). The D-Score varied across the three *Trp53*-null subtypes, with the p53null-Claudinlow^Ex^ subtype being the lowest, the p53null-Basal^Ex^ being intermediate, and the p53null-Luminal^Ex^ being the highest (*P*<0.05) (Fig. 1B); even though the p53null-Luminal^Ex^ subtype had the highest D-Score among the three p53-null subtypes, its score was still relatively intermediate when compared across the entire diverse murine tumor dataset. This indicates that, although the p53null-Luminal^Ex^ class is the most ‘luminal’ of the three *Trp53*-null classes, these tumors do not have as strong an association to normal mammary gland MatureLum cells as do murine MMTV-Neu tumors and human luminal A/B tumors (Pfefferle et al., 2015).

Taken together (Fig. 1 and Fig. S1), these results indicate that p53null-Basal^Ex^ and p53null-Claudinlow^Ex^ tumors are best considered murine counterparts for human basal-like and claudin-low subtypes, respectively. On the other hand, p53null-Luminal^Ex^ tumors uniquely contained traits of multiple human intrinsic subtypes, namely all three aggressive human subtypes (i.e. basal-like, HER2-enriched and luminal B). This finding suggests that p53null-Luminal^Ex^ tumors might be useful for studying aspects of several human subtypes.

### Profiling of secondary genetic aberrations highlights conserved DNA copy-number changes between human and murine *Trp53*-null tumors

**Fig. 2.**
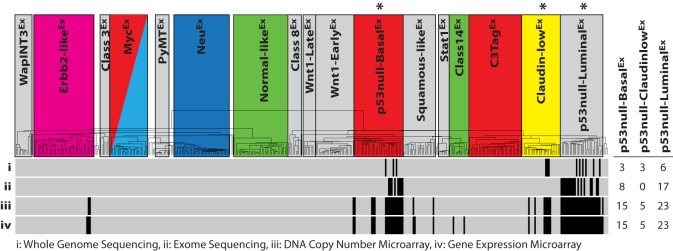
**Murine *Trp53*-null tumor datasets.** Sequencing and microarray technologies were used to produce four *Trp53*-null tumor datasets of varying sizes: (i) whole genome sequencing (*n*=12), (ii) exome sequencing (*n*=25), (iii) DNA copy-number microarray (*n*=43) and (iv) gene expression microarray (*n*=43). The intrinsic class of each sample is displayed on the dendrogram, with colored boxes being previously identified human subtype counterparts (Pfefferle et al., 2013). The hierarchical clustering location of each p53-null tumor within the datasets is displayed as a vertical black strip. *The *Trp53*-null transplant model produces heterogeneous tumors that primarily develop into one of these three murine expression subtypes. For each dataset, the number of tumors studied from each of the three murine classes highlighted by ‘*’ is displayed on the right-hand side of the figure.

In broad terms, disruption of normal *Trp53* signaling leads to an unstable genome due to a decreased ability to properly respond to the presence of genetic aberrations (Bieging et al., 2014). This phenotype leads to the accumulation of both small-scale mutations (e.g. insertions, deletions) and large-scale chromosomal rearrangements (e.g. translocations, copy-number variations) throughout the genome. Specific genetic aberrations are predicted to be responsible for determining a cell's subtype fate during tumorigenesis, but identifying these specific drivers has been challenging. Here, we leveraged the power of multiple ‘omic’ technologies to interrogate the secondary genetic aberrations underlying 43 different, independently arisen *Trp53*-null transplant tumors (Table S1). Specifically, Illumina sequencing and microarray technologies were used to produce four datasets of varying sizes: DNA whole-genome sequencing (*n*=12), DNA exome sequencing (*n*=25), DNA copy-number microarray (*n*=43) and gene expression microarray (*n*=43) (Fig. 2). For the purposes of our analyses, the DNA whole-genome and exome sequencing datasets were combined into a single dataset by reducing the whole genome samples to exome space, with all data deposited into the Sequence Read Archive (SRA).

**Fig. 3.**
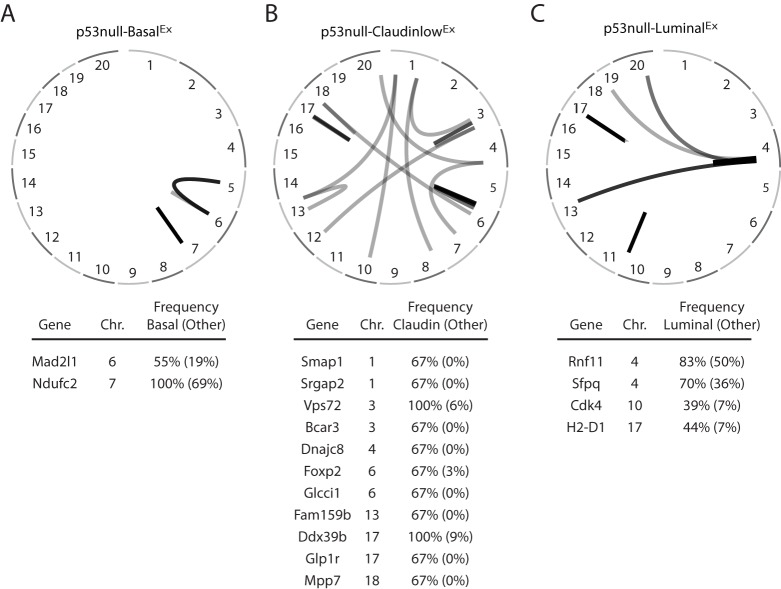
**Chromosome structural-variation analysis.** Displayed are circos plots of the structural variants (SVs) enriched within (A) p53null-Basal^Ex^, (B) p53null-Claudin-low^Ex^ and (C) p53null-Luminal^Ex^ tumors as determined by a two-class (class *x* versus all others) Fisher's exact test. The genes affected by these SVs are listed under each circos plot.

Given our hypothesis that the accumulation of specific secondary genetic events during tumorigenesis drives the development of specific tumor subtypes, we designed our statistical analyses to identify those genetic events that were enriched within a specific *Trp53*-null transplant classes as compared to the other two. Using this approach with our combined DNA whole-genome and exome dataset (Table S2), there were no identifiable genes that were enriched with non-silent somatic mutations within the p53null-Basal^Ex^ or p53null-Luminal^Ex^ classes, and only one gene in the p53null-Claudin-low^Ex^ class (*Dchs2*, *P*=0.005) when using a two-class (class *x* versus all others) Fisher's exact test (Fig. S2). We therefore focused on large-scale chromosomal rearrangements, amplifications and deletions as possible drivers of the murine *Trp53*-null subtypes. An analysis of chromosome structural variants (SVs) (Table S3) identified several rearrangements that were enriched within each of the three *Trp53*-null transplant classes using a two-class (class *x* versus all others) Fisher's exact test (Fig. 3). For instance, *Mad2l1* was enriched for SVs in p53null-Basal^Ex^ tumors. Mad2l1 plays an important role during metaphase by preventing progression into anaphase until all chromosomes are properly aligned (Schvartzman et al., 2010). Overexpression of *Mad2l1*, as is the case in p53null-Basal^Ex^ tumors (FDR 0%), might promote tumor development by further decreasing chromosome stability (Sotillo et al., 2007). Although these SVs that are enriched in certain *Trp53*-null transplant classes are intriguing, their biological effect on the tumor phenotype is unknown at this time. As a result, we were unable to definitively call any of these SVs drivers of these classes.

**Fig. 4.**
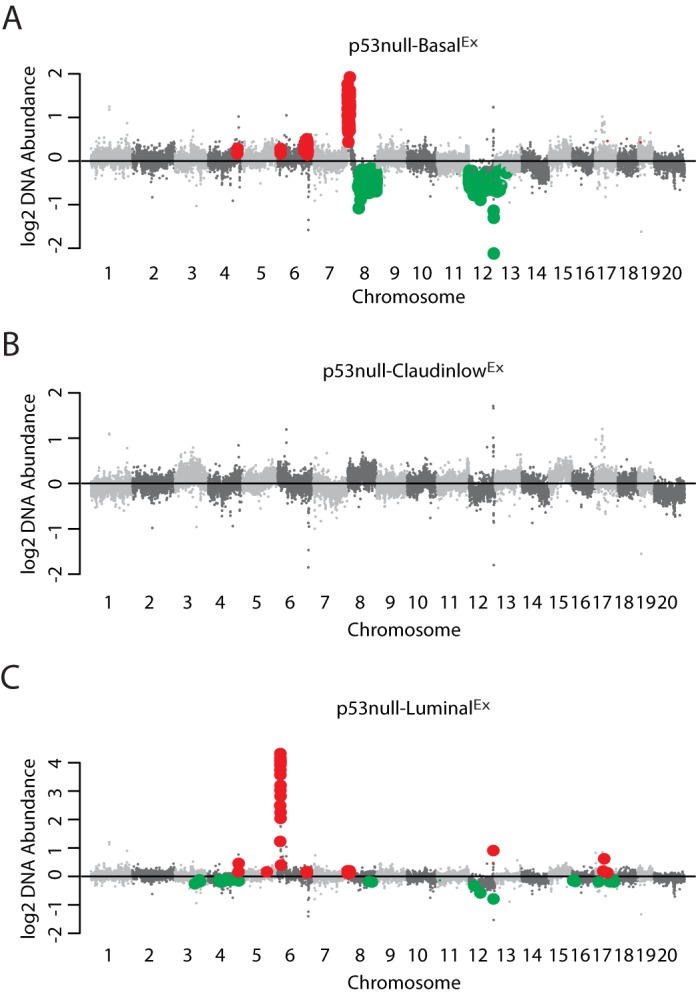
**DNA copy-number analysis.** Displayed in genomic order are the median class DNA copy-number levels for (A) p53null-Basal^Ex^, (B) p53null-Claudin-low^Ex^ and (C) p53null-Luminal^Ex^ tumors. DNA copy-number changes enriched within each of the three *Trp53*-null transplant classes were identified using a two-class (class *x* versus all others) SAM analysis. Genomic regions of significant gain are labeled in red and regions of significant loss are labeled in green.

The mechanism by which DNA copy-number variation leads to changes in the tumor phenotype, however, is more intuitive and more easily tested; therefore, we focused our attention on DNA amplifications and deletions as secondary genetic drivers. We were primarily interested in identifying genes in which the DNA copy-number variation was highly correlated with their gene expression, because this observation is consistent with causality. First, we identified DNA copy-number changes that were statistically enriched within each of our three *Trp53*-null transplant classes using a two-class (class *x* versus all others) SAM analysis (Fig. 4). Interestingly, both the p53null-Basal^Ex^ (Fig. 4A) and p53null-Luminal^Ex^ (Fig. 4C) classes had distinct genomic regions of DNA gains and losses, whereas the p53null-Claudin-low^Ex^ (Fig. 4B) class was relatively copy-number neutral, having no genomic regions enriched with gains or losses; these results are consistent with human studies that have highlighted several DNA copy-number events specific to basal-like tumors but few, if any, events in claudin-low tumors (Weigman et al., 2012). Specifically, p53null-Basal^Ex^ tumors were defined by both gains and losses on chromosome 8 and almost a complete loss of chromosome 12 (Fig. 4A), whereas p53null-Luminal^Ex^ tumors were defined by a DNA amplification on chromosome 6 (Fig. 4C).

**Fig. 5.**
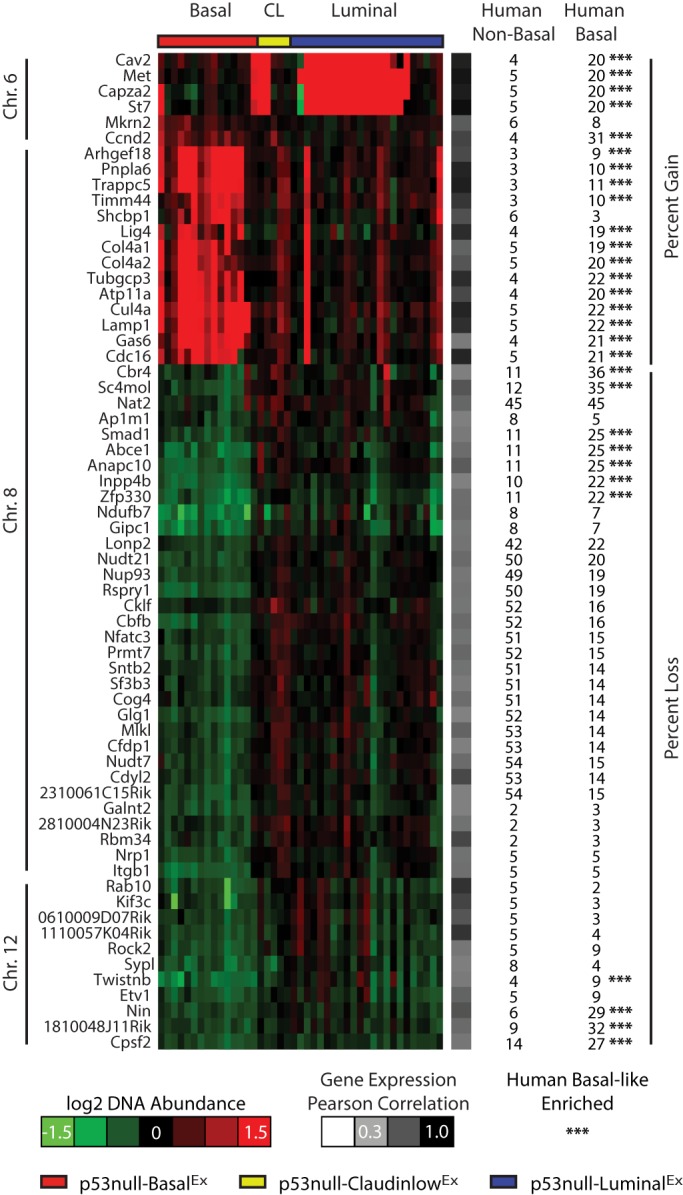
**Conserved DNA aberrations with human basal-like tumors.** Pearson correlations between DNA copy number and gene expression were determined for all genes within the significant regions of gain and loss from Fig. 4. Genes with a correlation greater than or equal to 0.5 are displayed in genomic order. The heatmap corresponds to DNA copy-number abundance. The percentage of human non-basal and human basal-like tumors affected by amplification or deletion of these genes is displayed. Human basal-like enriched events (indicated by ***) were determined using a two-class Fisher's exact test (*P*<0.05). CL, p53null-Claudin-low^Ex^.

To narrow the list of potential genetic drivers within these subtype-enriched regions, the Pearson correlation between DNA copy number and gene expression was calculated to highlight the genes that were the most concordant with DNA gains and losses (Fig. 5). A Pearson correlation cut-off of 0.5 or greater was used to define genes with a strong DNA-RNA association. A number of interesting genes fell into this classification. As we previously noted (Herschkowitz et al., 2012), *Inpp4b* (Pearson=0.5), a regulator of PI3K-AKT signaling (Agoulnik et al., 2011), was lost in p53null-Basal^Ex^ tumors on chromosome 12, similar to human basal-like tumors (Weigman et al., 2012). *Cul4a*, which is located on the p53null-Basal^Ex^ chromosome 8 amplicon, had a very high correlation with its gene expression (Pearson=0.86). CUL4A is a scaffolding protein for E3 ubiquitin ligase, which helps regulate the cellular concentration of key protein substrates, including CHK1, E2F1, ER-α and pol η, to name a few (Jackson and Xiong, 2009). Given the wide variety of cellular phenotypes that these protein substrates influence, such as proliferation and DNA repair (Jackson and Xiong, 2009), CUL4A has been proposed as a possible cancer driver and drug target (Sharma and Nag, 2014). The p53null-Luminal^Ex^ tumors had a distinct amplification of chromosome 6 (Fig. 4) and, within this region, four genes had high correlation with their gene expression levels (Fig. 5), including *Met* (Pearson=0.92). *Met*, which is also spontaneously amplified in a subset of *Brca1*/*Trp53* (Smolen et al., 2006) and *lunatic fringe* deleted (Xu et al., 2012) mouse mammary tumors, is a receptor tyrosine kinase for hepatocyte growth factor that regulates a variety of downstream signal transduction pathways, including MAPK and PI3K-AKT (Gastaldi et al., 2010).

Given that p53null-Basal^Ex^ tumors are counterparts for the human basal-like subtype (Pfefferle et al., 2013) and that p53null-Luminal^Ex^ tumors show features of all three aggressive human subtypes (i.e. basal-like, HER2-enriched and luminal B), we investigated The Cancer Genome Atlas (TCGA) DNA copy-number dataset to determine whether these spontaneous murine genetic events were also enriched in human basal-like breast tumors to highlight conserved features across species (Cancer Genome Atlas Network, 2012). Using a two-class (human basal-like versus human non-basal) Fisher's exact test, we identified 29 DNA copy-number gains or losses commonly enriched in human basal-like tumor genomes (*P*-value<0.05), which included gains of *Met* and *Cul4a* and loss of *Inpp4b* (Fig. 5).

### 2250L (p53null-Luminal^Ex^) tumors completely regress with crizotinib treatment

**Fig. 6.**
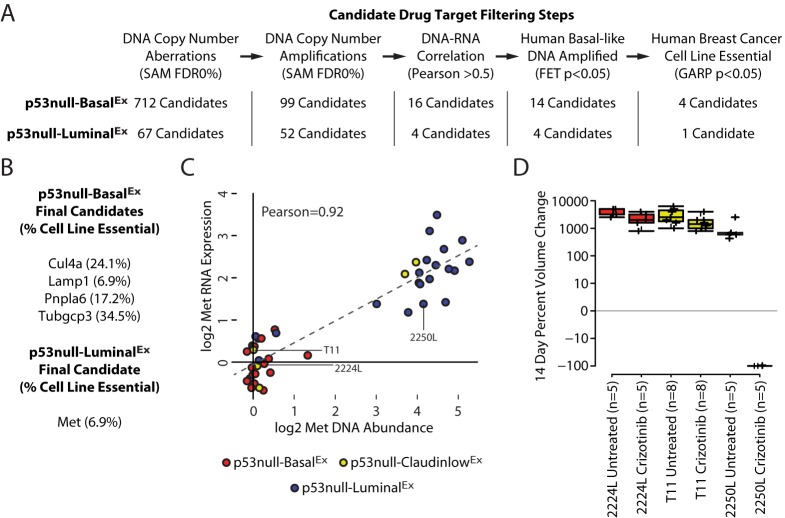
**Candidate drug targets.** (A) Candidate-drug-target filtering steps. (B) Final drug-target candidates for p53null-Basal^Ex^ and p53null-Luminal^Ex^ tumors. (C) Correlation of DNA and RNA for *Met*. (D) Change in tumor volume after 14 days of continuous crizotinib treatment displayed as box-and-whisker plots.

Although the identification of conserved genetic events helps to inform the molecular etiology of breast cancer, we were particularly interested in identifying genetic drivers that were also drug targets. Because conserved copy-number-amplified and overexpressed genes might also serve as targets against human basal-like breast tumors, we focused our attention on the genomic amplifications because their protein products are more directly targetable than the loss of protein function caused by deletions. Using this filtering step, we were able to limit our candidate drug target list to 14 genes for p53null-Basal^Ex^ tumors and four genes for p53null-Luminal^Ex^ tumors (Fig. 6A). Because many of these genes are located on the same amplified DNA segment, we hypothesize that specific genes within these segments are responsible for driving tumorigenesis and the others are passenger aberrations. To narrow the potential drug target list further, we used genome-wide pooled short-hairpin RNA (shRNA) dropout signature profiles obtained from the Donnelly – Princess Margaret Screening Centre (DPSC) Cancer Database (Marcotte et al., 2012; Koh et al., 2012) for 29 human breast cancer cell lines (Parker et al., 2009; Prat et al., 2013). This dataset was constructed by targeting ∼16,000 genes with lentiviral shRNA constructs to determine which genes are essential for cancer cell survival and proliferation (Marcotte et al., 2012; Koh et al., 2012). For our purposes, we used their *in vitro* breast cancer cell-line experiments to validate our conserved drug candidates from Fig. 5. Genes were considered essential if they were required for cell-line survival and proliferation in greater than 5% of the cell lines as defined by a Gene Activity Ranking Profile (GARP) *P*-value<0.05 (Marcotte et al., 2012; Koh et al., 2012). This relatively low cut-off was designed to identify both personalized and universal tumor drug targets. Using this final filtering step (Fig. 6A), we were able to highlight four potential essential genes for p53null-Basal^Ex^ tumors (*Cul4a*, *Lamp1*, *Pnpla6* and *Tubgcp3*) and one potential essential gene for p53null-Luminal^Ex^ tumors (*Met*) (Fig. 6B).

The fact that *Met* was the only candidate remaining after our filtering steps is particularly interesting given that genetically engineered loss of *Trp53* and overexpression of *Met* synergize to induce murine mammary tumors (Knight et al., 2013). These genetically engineered tumors were also responsive to crizotinib (Knight et al., 2013), a US Food and Drug Administration (FDA)-approved therapy for non-small-cell lung cancer (Solomon et al., 2014) that inhibits *Met* and *Alk* (Shaw et al., 2011). *Met* DNA amplification and expression had a bimodal correlation profile in our dataset, with p53null-Luminal^Ex^ tumors preferentially having this genetic aberration (Fig. 6C). Interestingly, a subset p53null-Claudin-low^Ex^ tumors were also *Met*-amplified, consistent with a previous observation that Met synergizes with p53 loss to promote tumors with claudin-low features (Knight et al., 2013). To formally test whether *Met* was a personalized drug target in our *Met-*amplified murine tumors, we treated three *Trp53*-null transplant tumor lines with crizotinib. Whereas neither 2224L (p53null-Basal^Ex^) nor T11 (p53null-Claudin-low^Ex^) tumors responded to treatment (noting that both of these lines were not *Met*-amplified), all of the 2250L (p53null-Luminal^Ex^) tumors had complete regression at the end of the 14-day treatment period (Fig. 6D). Unfortunately, we were unable to test whether either of the *Met-*amplified p53null-Claudin-low^Ex^ tumors were sensitive to crizotinib because they were not developed into transplantable tumor lines at the time of initial sample collection. Given the dynamic response of the 2250L (p53null-Luminal^Ex^) tumors, we hypothesize that the two *Met-*amplified p53null-Claudin-low^Ex^ tumors could have also been crizotinib-responsive. Because *Alk* was not differentially expressed across the three *Trp53*-null transplant classes, we propose that this dynamic response is due to differences in *Met* signaling, and, thus, spontaneous *Met* amplification is a driver of tumorigenesis in this mouse model of human breast cancer.

## DISCUSSION

Although increased public awareness and a greater understanding of tumor biology have led to improved patient survival rates, breast cancer is still the second leading cause of cancer related deaths in American women and TNBC has the worst prognosis at present (Cancer Genome Atlas Network, 2012; Curigliano and Goldhirsch, 2011). With so many patients either not responding or relapsing with the current standard of care, the molecular mechanisms underlying breast cancer are under intense investigation to identify new, personalized drug targets (Curigliano and Goldhirsch, 2011; Curigliano, 2012). This is especially true for TNBCs (basal-like and claudin-low), for which targeted treatment options remain an important unmet clinical need. Murine models provide an excellent resource for identifying genetic drug targets by highlighting conserved features between species (Pfefferle et al., 2013). Given that somatic *TP53* mutations are the most common genetic event in human TNBC (Cancer Genome Atlas Network, 2012), the *Trp53*-null mammary transplant mouse model is particularly useful for studying the etiology of TNBCs because almost all tumors that arise in this model are ER-negative, progesterone receptor (PR)-negative and not HER2-amplified.

The *Trp53*-null transplant model produces heterogeneous tumors that can be classified into three major subtypes/classes based on gene expression profiles: p53null-Basal^Ex^, Claudin-low^Ex^ and p53null-Luminal^Ex^ (Pfefferle et al., 2013); we do note that for each transplant line, the subtype and genetics are consistent over time and over multiple passages (Herschkowitz et al., 2012). Using gene expression comparisons, we show that p53null-Basal^Ex^ tumors are counterparts for the human basal-like subtype, whereas p53null-Claudin-low^Ex^ tumors are counterparts for the human claudin-low subtype. Although p53null-Luminal^Ex^ tumors have features of human luminal tumors (i.e. HER2-enriched and luminal B), these tumors had intermediate ‘differentiation scores’ (D-Scores), similar to luminal progenitor cells (Prat et al., 2010). The original nomenclature for this class was derived by an observed association to the human luminal subtypes based on a few luminal markers (Herschkowitz et al., 2012), but more recent work has shown that mature luminal and luminal progenitor cells share many of the same features (Pfefferle et al., 2015; Kannan et al., 2013). For instance, both mature luminal and luminal progenitor cell fluorescence-activated cell sorting (FACS) populations are EpCAM-positive (EpCAM^pos^) and Krt18^pos^ (Lim et al., 2009; Kannan et al., 2013), indicating that broader analyses are required to distinguish between these cell types within tumors. These findings help explain why p53null-Luminal^Ex^ tumors were found to also have traits in common with the basal-like subtype, because human basal-like tumors share features of luminal progenitor cells (Pfefferle et al., 2015; Lim et al., 2009). These observations indicate that p53null-Luminal^Ex^ tumors share features with all three poor-outcome human subtypes (i.e. basal-like, HER2-enriched and luminal B), including TP53 loss (Cancer Genome Atlas Network, 2012).

Once these human-to-murine subtype counterparts were defined, secondary-genetic-aberration profiling was performed to identify candidate drivers of tumorigenesis. Through the use of a comparative genomics analysis, we identified a number of murine mammary subtype-specific copy-number events in our *Trp53*-null transplant murine model, which mimicked similar events in human basal-like tumors. We were particularly interested in genes that had a high correlation between their copy number and gene expression because, for these cases, we propose that the copy-number change is the mechanism that directly influences the expression of the genes within those genomic regions. We propose that a subset of these conserved features might serve as effective drug targets against human basal-like tumors. To highlight this subset of genes, we utilized genome-wide pooled shRNA dropout signature profiles obtained from the DPSC Cancer Database (Marcotte et al., 2012; Koh et al., 2012) for 29 human breast cancer cell lines (Parker et al., 2009; Prat et al., 2013). Through these various filtering steps, we were able to identify four potential driver genes that were DNA-amplified in p53null-Basal^Ex^ tumors. Although additional experiments will be required to validate that these genes are viable drug targets for this tumor subtype as they are for breast cancer cell lines (Marcotte et al., 2012; Koh et al., 2012), we propose that *Cul4a* is an attractive candidate because *CUL4A* amplification and overexpression have been observed in the human basal-like breast cancer subtype and were demonstrated to drive tumorigenesis both *in vitro* and *in vivo* (Chen et al., 1998; Gupta et al., 2002; Saucedo-Cuevas et al., 2014). By contrast, *Met* was the only candidate driver gene of p53null-Luminal^Ex^ tumors. This class had a reproducible DNA amplification of wild-type *Met*, suggesting that this could be a driver for this murine subtype. Crizotinib treatment resulted in complete tumor regression at the end of the 14-day treatment period in our 2250L (p53null-Luminal^Ex^) tumor. MET is an important receptor tyrosine kinase that can activate a variety of signal transduction pathways, including MAPK and PI3K-AKT. These results suggest that *Met* is a driving oncogene in this murine model subtype, and that crizotinib or similar MET-targeted therapies might be effective against human basal-like tumors that contain *Met* amplification. These experimental results are particularly relevant given that about 20% of human basal-like tumors have amplification of *MET*. It is important to note, however, that identifying effective single-agent therapies is inherently difficult owing to the presence of resistant subpopulations within the tumor and/or due to molecular mechanisms that allow the tumor to evade treatment, such as kinome reprogramming (Duncan et al., 2012). Although not directly tested here, we propose that crizotinib-responsive tumors would eventually develop resistance and, therefore, a drug combination would most likely be needed to produce the best long-term outcome (Roberts et al., 2012b).

Although several clinical trials have been designed to test the efficacy of anti-MET therapy in breast cancers (Ho-Yen et al., 2015), early results suggest that they do not offer additional clinical benefit to the current standard of care in patients with TNBC (Diéras et al., 2015). We propose that these negative results were a reflection of the overall clinical trial design in which TNBC patients were not additionally stratified by *MET*-amplification status. This hypothesis is supported by a non-small-cell lung cancer clinical trial, which found that MET inhibition only added clinical benefit in the MET-positive subpopulation of patients (Spigel et al., 2013). Similar companion diagnostics should be considered to further stratify TNBC patients into those with MET amplification/signaling to better identify those patients most likely to respond to anti-MET therapy.

In conclusion, our work highlights the importance of comparative genomic studies as a preclinical tool for discovering personalized drug targets and for determining which patient populations are most likely to respond to treatment. The identification of these drug targets is especially important for TNBC, in which tumor heterogeneity suggests that a universal drug target for this subtype is unlikely. Given the wealth of mouse models and genomic data available for breast cancers, we propose that these types of studies should be an integral part of early phase drug development to expedite the development of more personalized treatment regimens.

## MATERIAL AND METHODS

### *Trp53*-null transplant model

The BALB/c *Trp53*-null mouse model used in this study was created using a germline mutation that produced a truncated and nonfunctional version of Trp53 (Jacks et al., 1994). *Trp53*-null mice, however, are predisposed to developing a variety of different tumor types, such as lymphomas, which occur prior to breast tumor development (Jacks et al., 1994). To study p53 signaling in breast cancer, a transplant model was developed in which the mammary pads of 8- to 10-week-old female *Trp53*-null mice were transplanted into the cleared mammary pads of 21- to 24-day-old wild-type BALB/c female recipient mice (Jerry et al., 2000). These mice were then monitored until breast tumor development. A few of these samples were frozen during tumor collection so that they could be used for orthotopic, syngeneic transplant experiments following an initial characterization of the primary tumor (Usary et al., 2013; Roberts et al., 2012b). It is important to highlight that these individual tumor lines (e.g. 2224L, T11 and 2250L) differ from traditional cell lines in the fact that they are never grown *in vitro* but are rather always grown *in vivo* through tumor transplantations from one recipient mouse to another.

### Gene expression

Microarray gene expression data from 27 murine models of mammary carcinoma, and normal mammary tissue, were downloaded from the following gene expression omnibus (GEO) entries: GSE3165, GSE8516, GSE9343, GSE14457, GSE15263, GSE17916, GSE27101 and GSE42640 (Pfefferle et al., 2013). The 385-sample dataset was normalized to correct for microarray platform bias as previously described (Pfefferle et al., 2013).

Tumor differentiation scores (D-Scores) were calculated across the microarray dataset as previously described (Prat et al., 2010). Gene expression signatures were created for the three murine classes enriched with the *Trp53*-null transplant model (p53null-Basal^Ex^, Claudin-low^Ex^ and p53null-Luminal^Ex^) by performing a two-class (class *x* versus all others) Significance Analysis of Microarrays (SAM) analysis on the microarray dataset (Tusher et al., 2001). Signatures were defined as all genes highly expressed in the class of interest with a false discovery rate (FDR) of 0%. Similarly, pathway signatures were created as previously described (Pfefferle et al., 2013). Expression scores for each gene and pathway signature were determined by calculating the mean expression of the signature within each sample in the UNC308 (Prat et al., 2010), Combined855 (Harrell et al., 2012) and Metabric (Curtis et al., 2012) human breast cancer datasets.

### Whole-genome and exome sequencing

Illumina libraries were constructed with 1 mg of genomic DNA according to the manufacturer's protocol with the following modifications: (1) DNA was fragmented with a Covaris E220 DNA Sonicator to size range between 100 and 400 bp, (2) Illumina adaptor-ligated library fragments were amplified in four 50-ml PCR reactions for 18 cycles, and (3) solid-phase reversible immobilization bead cleanup was used for enzymatic purification throughout the library process, as well as final library size selection targeting 300-500 bp fragments. For whole-genome sequencing, each library was sequenced on four lanes of an Illumina HiSeq2000 instrument (2×101 bp) per the manufacturer's recommendations for an average of 65.7 Gb per sample (∼19× haploid coverage). Reads were mapped to the Mm9 reference sequence by BWA v.0.5.912 with the following parameters: -t 4 -q 5. Alignments were merged and duplicates marked by Picard v.1.46. For exome sequencing, individually barcoded libraries were pooled (three pools of ten samples and one pool of five samples) prior to capture with the Agilent mouse whole-exome kit. Following capture, pools were sequenced on two lanes (one lane for the five-sample pool) of an Illumina HiSeq2000 instrument (2×101 bp). The DNA whole-genome and exome sequencing datasets were combined into a single dataset by reducing the whole-genome samples to exome space. All sequencing data was uploaded to the Sequence Read Archive (SRA) under accession number SRP061710.

### Somatic mutation and SV detection

We performed comprehensive somatic mutation detection with the whole-genome sequencing/exome data for each tumor sample using the BALB/c data as a matched ‘normal’ control. Point mutations [single-nucleotide variants (SNVs)] were called using SomaticSniper v0.7.3 and VarScan v2.2.6, with the union of both callsets filtered to remove false positives as previously described (Koboldt et al., 2012). SNVs that overlapped mouse dbSNP 128 positions or that clustered at densities greater than 2 per 500 kb were removed as likely germline variants. Somatic indels were called using the Genome Analysis ToolKit (GATK-1.0.5336). Somatic structural variants (SVs) were called using BreakDancer v1.2 followed by *de novo* assembly with TIGRA. Somatic SNVs, indels and SVs were annotated using an in-house pipeline with transcripts from Ensembl 58 and NCBI build 37.

### Analysis of somatic mutations and SVs

SNVs were collapsed to a gene level so that all non-silent somatic mutations affecting the same gene were treated equally regardless of the actual mutation. A two-class (class *x* versus all others) Fisher's exact test was performed to identify genes preferentially mutated within each *Trp53*-null class (*P*-value<0.05). Somatic SVs were collapsed to a gene level so that all SVs affecting the same gene were treated equally regardless of the actual SV. Genes were defined as being affected by the SV if the start or end of the SV occurred within the RefSeq gene region. A two-class (class *x* versus all others) Fisher's exact test was performed to identify genes preferentially affected by SVs within each *Trp53*-null class (*P*-value<0.05). Because all of the p53null-Claudin-low^Ex^ tumors were analyzed using whole-genome sequencing, a second two-class Fisher's exact test was performed on these tumors, in which only the 13 whole-genome profiled tumors were included in the analysis to reduce the likelihood of the p53null-Claudin-low^Ex^-enriched SVs being an artifact of methodology.

### DNA copy number

DNA array comparative genomic hybridization (aCGH) data were downloaded for the *Trp53*-null transplant tumors classified as p53null-Basal^Ex^, Claudin-low^Ex^ or p53null-Luminal^Ex^ by gene expression profiling from GEO entry GSE27101 (Herschkowitz et al., 2012). In addition, genomic DNA was extracted from five *Trp53*-null transplant tumors using a DNeasy blood and tissue kit (Qiagen #69504), labeled with a Sure Tag DNA kit (Agilent #5190-4240), and hybridized to 244K CGH microarrays (Agilent #G4415A) as previously described (Herschkowitz et al., 2012). DNA aCGH data was uploaded to GEO under accession number GSE71071.

The 43-sample aCGH dataset was extracted from the University of North Carolina (UNC) Microarray Database as log_2_ Cy5/Cy3 ratios, filtering for probes with Lowess normalized intensity values greater than ten in the control channel and for probes with data on greater than 70% of the microarrays (Herschkowitz et al., 2012). The probes that passed these filters were then oriented in genomic order and a ten-probe average was calculated on consecutive groups of ten probes across each chromosome, resulting in a final dataset of 23,181 features. A two-class (class *x* versus all others) SAM analysis was performed to identify genomic regions of amplification or deletion unique to each class (FDR of 0%).

Level-3 DNA segmentation data was downloaded from The Cancer Genome Atlas (TCGA) data portal for 715 breast cancer samples (Cancer Genome Atlas Network, 2012). Genomic regions of amplification and deletion were defined as having a log_2_ segmentation value greater than 0.3 or less than −0.3, respectively. Genes preferentially amplified or deleted in the human basal-like subtype were determined using a two-class (human basal-like versus human non-basal) Fisher's exact test (*P*-value<0.05).

### Genes essential for the survival and proliferation of human breast cancer cell lines

Genome-wide pooled shRNA dropout signature profiles were obtained from the Donnelly – Princess Margaret Screening Centre (DPSC) Cancer Database (Marcotte et al., 2012; Koh et al., 2012) for 29 human breast cancer cell lines. Genes were considered essential if they were required for cell-line survival and proliferation in at least 5% of cell lines as defined by a Gene Activity Ranking Profile (GARP) *P*-value<0.05.

### Crizotinib treatment

All mouse work was performed under protocols approved by the UNC Institutional Animal Care and Use Committee. Of the 43 *Trp53*-null transplant tumors used in this study, only four primary tumors were available as tumor transplant lines for follow-up passaging experiments: 2224L (p53null-Basal^Ex^), 2225L (p53null-Basal^Ex^), T11 (p53null-Claudin-low^Ex^) and 2250L (p53null-Luminal^Ex^). A single representative tumor from each *Trp53*-null transplant subtype was randomly selected for crizotinib treatment experiments. One-million tumor cells were suspended in Matrigel and injected subcutaneously into the lower-right mammary pad of BALB/c wild-type female mice so that each mouse developed a single tumor. When tumors were approximately 8 mm by 8 mm in size, mice were randomized to either the crizotinib (ChemShuttle #877399-52-5) or untreated group. Crizotinib chow was made by OpenSource Diets to a final concentration of 50 mg/kg/day and was given continuously over the 14-day treatment period to monitor tumor growth. Tumor volume was calculated from two-dimensional measurements (Volume=[(width)^2^×length]/2). The percent change in volume at 14 days was used to quantify response.
